# Synthesis and Thermal Oxidation Resistance of Boron-Rich Boron–Carbide Material

**DOI:** 10.3390/ma16196526

**Published:** 2023-10-01

**Authors:** Seth Iwan, Wesley Sutton, Paul A. Baker, Raimundas Sereika, Yogesh K. Vohra

**Affiliations:** 1 Department of Physics, University of Alabama at Birmingham, Birmingham, AL 35294, USA; iwanseth@uab.edu (S.I.); pabaker@uab.edu (P.A.B.); rsereika@uab.edu (R.S.); 2 Department of Chemistry, Physics, and Astronomy, Georgia College and State University, Milledgeville, GA 31061, USA; wesley.sutton@bobcats.gcsu.edu

**Keywords:** boron–carbon system, thermal oxidation, extreme environments, mechanical properties, Raman spectroscopy

## Abstract

A boron-rich boron–carbide material (B_4+δ_C) was synthesized by spark plasma sintering of a ball-milled mixture of high-purity boron powder and graphitic carbon at a pressure of 7 MPa and a temperature of 1930 °C. This high-pressure, high-temperature synthesized material was recovered and characterized by X-ray diffraction, X-ray photoelectron spectroscopy, Raman spectroscopy, Vickers hardness measurements, and thermal oxidation studies. The X-ray diffraction studies revealed a single-phase rhombohedral structure (space group R-3m) with lattice parameters in hexagonal representation as *a* = 5.609 ± 0.007 Å and *c* = 12.082 ± 0.02 Å. The experimental lattice parameters result in a value of δ = 0.55, or the composition of the synthesized compound as B_4.55_C. The high-resolution scans of boron binding energy reveal the existence of a B-C bond at 188.5 eV. Raman spectroscopy reveals the existence of a 386 cm^−1^ vibrational mode representative of C-B-B linear chain formation due to excess boron in the lattice. The measured Vickers microhardness at a load of 200 gf shows a high hardness value of 33.8 ± 2.3 GPa. Thermal gravimetric studies on B_4.55_C were conducted at a temperature of 1300 °C in a compressed dry air environment, and its behavior is compared to other high-temperature ceramic materials such as high-entropy transition metal boride. The high neutron absorption cross section, high melting point, high mechanical strength, and thermal oxidation resistance make this material ideal for applications in extreme environments.

## 1. Introduction

The boron-rich boron–carbide materials are of interest because of their high strength and abrasion resistance applications, their high neutron absorption cross section in nuclear reactor control applications, and their incorporation in the fabrication of light-weight composites in body armor applications. One contributor to boron’s complexity is its propensity to waver between metallic or insulating properties, which arises due to frustration caused by the localization of its three valence electrons [1]. As of 2017, even boron’s most understood allotropes frequently have disagreeing theoretical predictions and experimental observations of their electronic properties [2]. One industry in which boron has a wide impact is the nuclear industry. ^10^B has a neutron cross section of 3840 barns, making it highly effective at capturing neutrons from fission chain reactions [3]. Once absorbed, the ^10^B nucleus decays into an alpha particle and a lithium nucleus [4], which are unable to continue the fission reaction. This makes ^10^B_4_C an excellent option for control rods used to adjust the rate of fission in a reactor, as well as neutron shielding in general. Boron-rich boron–carbon compounds could offer high-strength materials that, due to being mostly boron, still offer high neutron absorption rates.

Boron icosahedra (B_12_), a unique structure that forms in boron isotopes and compounds, has been studied extensively under ambient and high pressures [5,6]. In the boron–carbon system, B_50_C_2_ (3.8 atomic percent carbon) has been studied in detail and synthesized in a metastable state by a variety of methods [7,8,9]. For slightly higher atomic concentrations, approximately 8.8 atomic percent of carbon, it has been shown that boron icosahedra interconnects with tri-atom chains to form stable structures [10,11]. We are interested in the composition range between 8.8 atomic percent and 20 atomic percent carbon, where the rhombohedral B_4+δ_C phase is expected to be stable and whose thermal and mechanical properties can be investigated in detail.

This study establishes a method of direct synthesis of boron-rich boron–carbide material (B_4+δ_C) starting from precursor material containing high-purity boron powder mixed with graphite powder at high pressures and high temperatures. A single-phase rhombohedral structure has been synthesized in bulk and characterized by a variety of analytical techniques. The thermal oxidization in air has been characterized up to 1300 °C and compared with other high-temperature ceramic materials.

## 2. Materials and Methods

In the synthesis process, boron powder (99.99% purity from SkySpring Nanomaterials, Houston, TX, USA) and graphite powder (99.9995% purity from Thermo Scientific, Waltham, MA, USA) were mixed to have a 3.8% atomic concentration for the intended composition of the B_50_C_2_ compound. For the 3.8 atomic percent precursor, 7.0005 g of boron powder was mixed with 0.3112 g of carbon powder. The powders were loaded into a tungsten carbide canister with two 7-g tungsten carbide balls and 15 mL of acetone. The container was then loaded into a SPEX, Metuchen, NJ, USA, 8000M MIXER/MILL. The material was ball-milled for 6 h in 1-h segments, with 12 min of cool-down time between each to ensure the mill did not overheat. Once finished, the canister and material were placed open into a Thermo Scientific, Waltham, USA, lab-line vacuum oven at ambient temperature for 45 min to allow the residual acetone to evaporate. The material was then removed from the canister and put through a #400 sieve to ensure no large granules were left.

Spark plasma sintering (SPS) was used to sinter and synthesize the precursor powder once it was made. SPS was performed in a DR. FRITSCH, Fellbach, Germany, FAST/SPS-Sintering Press DSP 507. A five-part die system was used, consisting of an outer cylindrical mold and an interior cylindrical mold in two pieces, as well as a top and bottom punch. The outer cylindrical mold had partially drilled holes in the sides for later use with the SPS system’s pyrometer. The sample is wrapped in a molybdenum sheet and then covered with graphite foil. The molybdenum sheet was used to prevent contaminating the sample with graphite, and the graphite sheet was used to ease the removal of the molybdenum and sample from the mold after sintering was completed.

In each sample, either one or one and a half grams of material were loaded into the mold. The outside of the mold was surrounded by a graphite blanket to help thermally insulate it, with a hole cut in the blanket to allow the pyrometer to be aligned with the drilled hole in the side of the outer mold piece. A piece of graphite blanket was also used on the top of the mold, with a hole cut out for the upper punch. To protect the pistons in the SPS system, silicon–carbide plates were used on the top and bottom of the mold.

Once loaded, the sample was compressed to 3 MPa to confirm the mold would compress properly. The system was then sealed and evacuated, and nitrogen gas (95% N_2_, 5% H_2_) was pumped into the system at a rate of 20 L per minute. The sample was then compressed to 7 MPa and rapidly heated to its max temperature, between 1500 and 1930 °C. The average heating rate was 45 °C/min. The sample is then left for 10–20 min at max temperature before being cooled to 300 °C. The average cooling rate was 50 °C/min. Once cooled to 300 °C, the nitrogen gas was cut off and the chamber was purged. The sample was then removed from the chamber to finish cooling.

Polishing was used to clean the samples post-sintering. Once the sample was fully cooled and removed from the mold, the outer layers of molybdenum and graphite had to be removed. The graphite layer could be removed with a straight edge, while significant portions of the molybdenum sheet would have to be removed from the sample by polishing. The samples were polished using diamond grit sandpaper at 300 rpm. This was used to prepare the sample for XRD by flattening the top and bottom surfaces of the sample parallel to each other and making sure the sides were perpendicular to the top and bottom surfaces. Several samples were also further polished for microhardness testing. This was carried out by casting the samples in quick-cure epoxy with one surface of the sample exposed. The surface was then polished with increasing grit sandpaper to produce a smooth, flat surface. The grits used were 180, 320, 400, 600, 800, 1200, and 1500.

X-ray diffraction (XRD) was performed on the samples once polished. The samples were studied using powder XRD in an Empyrean X-ray Diffraction System, which was manufactured by Malvern Panalytical B.V., Almelo, Neverlands. The resulting XRD data was analyzed using High-Score plus (version 4.8) and GSAS II. The XRD patterns were collected on boron and graphite precursors as well as synthesized B_4+δ_C samples.

Thermal oxidization measurements were conducted during a thermogravimetric analysis (TGA) stage. The Linsies Robbinsville, USA, STA PT 1600(TGA-DSC) is capable of reaching a temperature of 1600 °C while controlling the atmospheric conditions inside the furnace. A type-k thermocouple was used to read the sample temperature inside the Al2O3 crucible. The type-k thermocouple is a long, thin alumina rod that lifts the crucible above the high-precision beam balance and into the furnace. As temperature changes, thermal expansion will cause the long stem to bend, resulting in a change in the center of mass, which is seen as a perceived mass change. This bending is repeatable and is accounted for by creating zero curves, which are made by cycling the TGA stage through the desired heating profile with the empty crucible repeated 3–5 times. These curves are then averaged together and subtracted from the actual measurement curve.

## 3. Results

**Figure 1:**
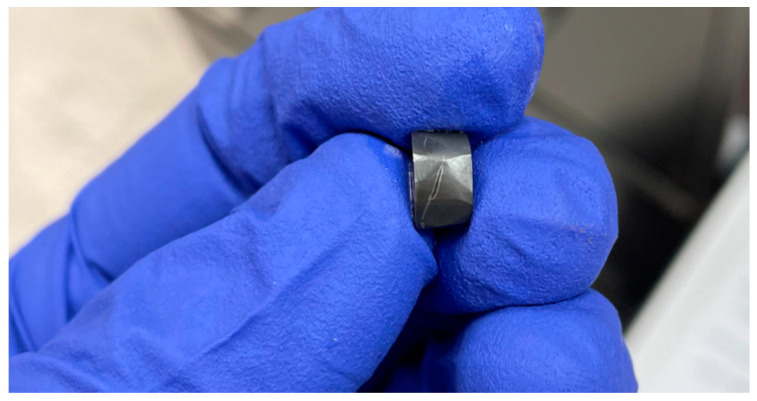
Handheld B_4+δ_C sample synthesized by the spark plasma sintering process. The image shown is during the polishing process, and the silver-colored streaks are from molybdenum foil surrounding the sample that still needs to be removed.

Figure 1 shows the B_4+δ_C sample synthesized at 7 MPa and 1930 °C and recovered from the sample assembly in the spark plasma sintering system.

**Figure 2:**
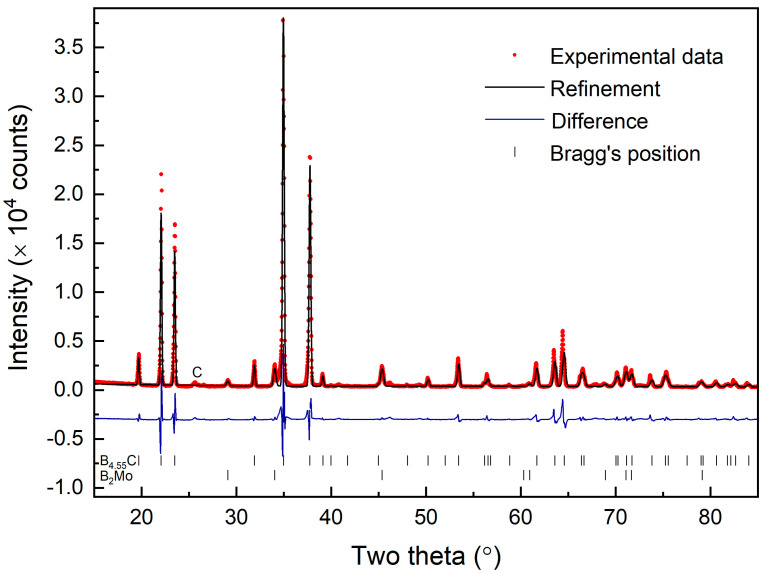
The measured X-ray diffraction pattern for the synthesized B_4+δ_C sample recorded with an X-ray wavelength of λ = 1.5406 Å. Rietveld refinement of the X-ray diffraction results in the assignment of a rhombohedral phase of B_4+δ_C with a lattice parameter described in the text. A very weak (3%) MoB_2_ phase was detected due to the small amount of impurity from the sample container in the synthesis process. A very low-intensity diffraction peak from free carbon in the sample is labeled as (C).

Figure 2 shows the measured X-ray powder diffraction pattern of the synthesized sample with an X-ray wavelength of λ = 1.5406 Å. Figure 2 also shows Rietveld refinement, resulting in a fit to the rhombohedral structure (space group R-3m) with lattice parameters in hexagonal representation as a = 5.609 ± 0.007 Å and c = 12.082 ± 0.02 Å (PDF#35-0798 from the JCPDS data base). There is also a small amount of MoB_2_ phase (approximately 3%) present in the Rietveld refinement resulting from the formation of molybdenum diboride between the sample powder and molybdenum foil during the spark plasma sintering process. The residual MoB_2_ is on the surface of the sample and can be removed with further polishing. The fitted lattice parameters of the MoB_2_ impurity phase are a = 3.038 Å and c = 3.068 Å.

**Figure 3:**
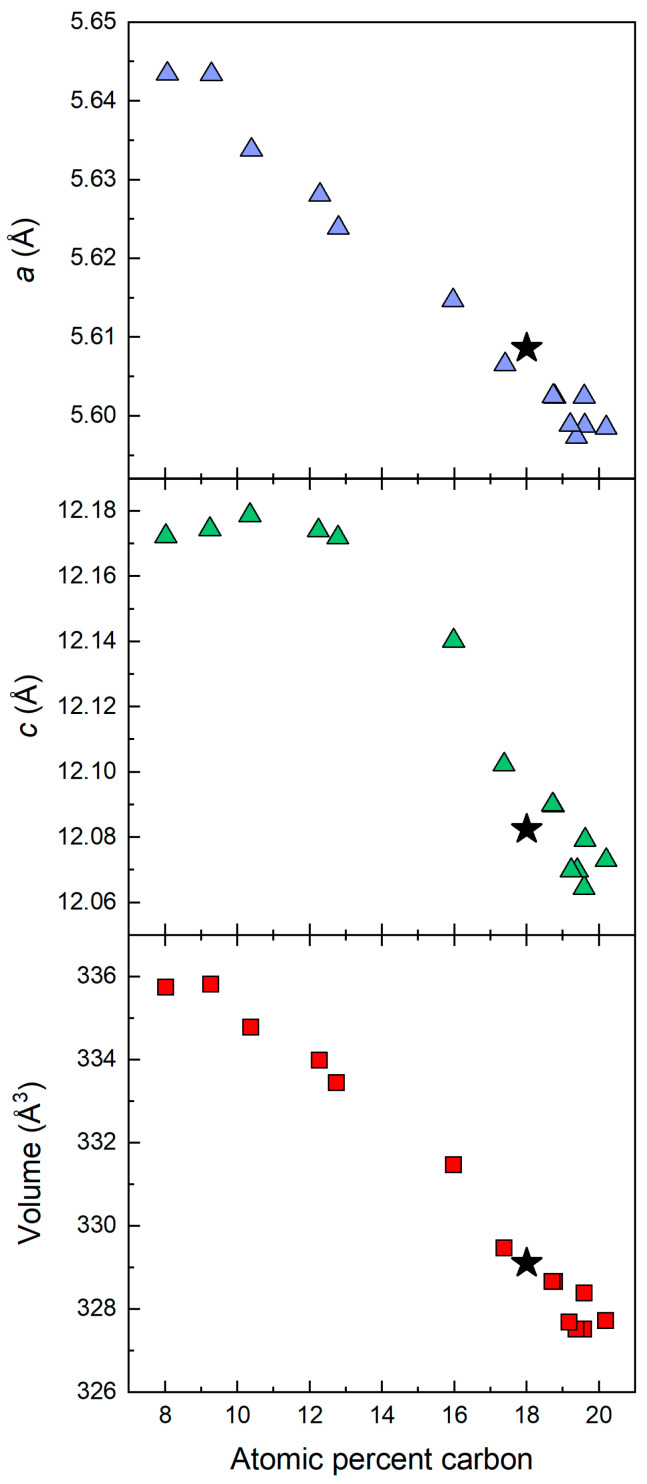
Our measured lattice parameters and unit cell volume for the rhombohedral phase of B_4+δ_C in the hexagonal representation marked by asterisk * is superposed on data from Ref. [12] between atomic percent carbon range of 8.8% and 20%. This superposition results in 18 atomic percent carbon or material composition B_4.55_C in our case.

The lattice parameters of the rhombohedral phase of B_4+δ_C compounds have been extensively studied in the range of carbon from 8.8 atomic percent to 20 atomic percent [12]. In Figure 3, we show our measured lattice parameters and unit cell superimposed on the data from Ref. [12]. From this superposition, we estimate our atomic concentration of carbon to be 18%, or the composition of the compound to be B_4,55_C. We started with 3.8 atomic percent carbon in the precursor material; however, molybdenum foil surrounding the sample serves as a sink of boron, and a carbon rich B_4,55_C phase is formed during the spark plasma sintering process. Such a determination is consistent with the Raman spectroscopy data described later in this paper.

**Figure 4:**
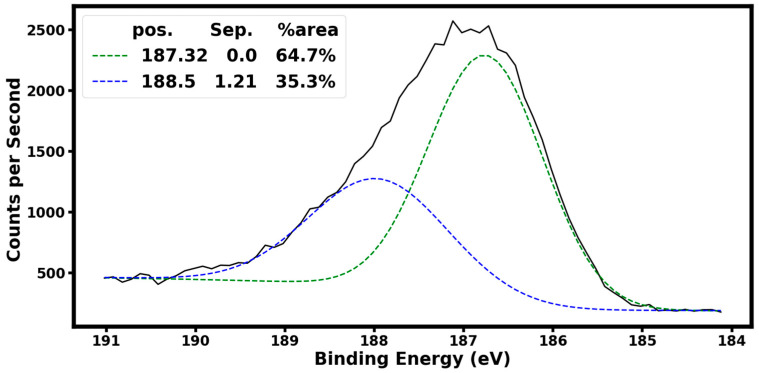
The measured X-ray photoelectron spectroscopy (XPS) high-resolution scan of the boron 1 s peak from the B_4,55_C sample showing a doublet structure with peaks at 188.5 eV (B-C bonding) and 187.32 eV (B-B) bonding.

Figure 4 shows X-ray photoelectron spectroscopy (XPS) of the B_4,55_C sample using a Phi Electronics, Inc., Chanhassen, USA, Versaprobe II equipped with a monochromatic Al X-ray source with a 100 µm spot size at 25 W. The high-resolution scan of the boron B1s peak was taken with a 0.1 eV step size and a pass energy of 23.5 eV. Our XPS analysis shows that 64.7% of the boron is B-B bonded (binding energy of 187.32 eV), and the remaining 35.3% is B-C bonded (binding energy of 188.5 eV). This B-C binding energy is consistent with the value of 188.6 eV reported for chemical states of boron B1s reported for boron–carbide in the literature [13].

**Figure 5:**
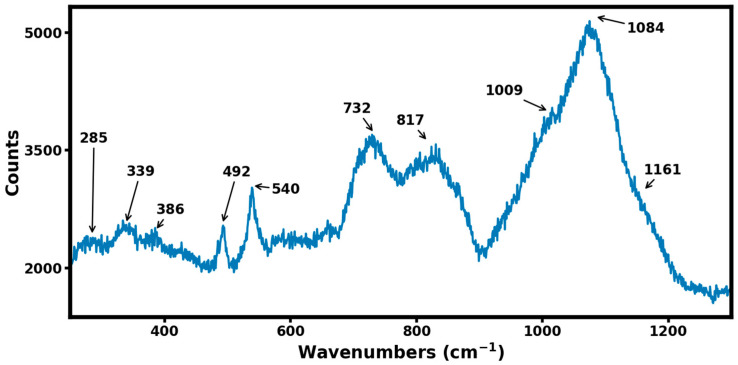
The Raman spectrum of the B_4,55_C sample recorded with a laser excitation of 532 nm. The Raman mode frequencies are indicated, and their assignments are discussed in the text.

Figure 5 shows the Raman spectrum from the B_4,55_C sample in the 200–1300 wavenumbers range recorded with a laser wavelength of 532 nm. Several high-frequency modes in the range of 700 cm^−1^ and 900 cm^−1^ are attributed to intra-icosahedral or inter-icosahedral B-B bonds, while the strong Raman band at 1084 cm^−1^ is attributed to the breathing mode of the icosahedron [14]. It should be noted that, in comparison to the Raman spectrum of stoichiometric B_4_C [14], Figure 5 shows additional Raman modes at 386 cm^−1^ and 1161 cm^−1^. These additional Raman modes are characteristic of boron-rich boron–carbide materials, as 1161 cm^−1^ mode is thought to occur from a change in the breathing mode of icosahedron due to excess boron, and 386 cm^−1^ mode is attributed to boron substitution, leading to the formation of the C-B-B linear chain in a boron-rich boron–carbide material.

**Figure 6:**
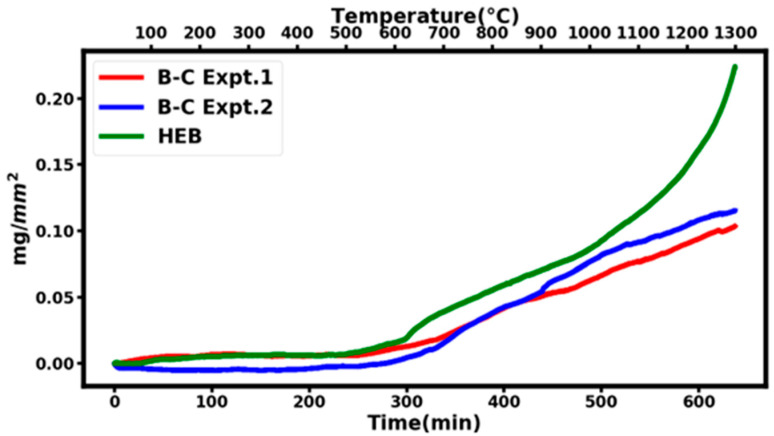
The measured increase in mass expressed as mg/mm^2^ of the B_4,55_C sample and a high-entropy boride (HEB) as a function of temperature up to 1300 °C. Both samples were heated at a constant rate of 2° per min, and mass gain was normalized to surface area for a quantitative comparison. The zero curve with an empty crucible has been subtracted from all the data.

The thermal oxidization resistance of the B_4,55_C sample was measured in compressed dry air at a temperature of 1300 °C. The previous high-temperature oxidation behavior of boron–carbide has been limited to a temperature of 1000 °C [15]. To have a precise comparison between various experimental runs on the B_4,55_C sample and a comparison with other high-temperature ceramics, all samples were sectioned into cubes so that their surface area could be measured, allowing the measured gain in mass to be normalized by surface area. Once the sample mass and surface area are recorded, the sample is loaded into the TGA furnace. Air is then evacuated from the furnace/balance chamber and then refilled with compressed dry air. Compressed dry air is slowly circulated through the furnace to replenish the oxygen supply. Figure 6 shows the results of two different experiments on B_4,55_C samples at 1300 °C labeled B-C Expt.1 (27.5 mg sample with a surface area of 33.27 mm^2^) and B-C Expt.2 (18 mg sample with a surface area of 27.45 mm^2^). The two experimental high-temperature runs are in reasonable agreement with each other, given the experimental uncertainties. Figure 6 also shows a similar thermal oxidation study at 1300 °C on a high-entropy boride (HEB) with a chemical composition of (HfMoNbTaZr)B_10._ It is important to note that the thermal oxidation resistance of the B_4,55_C sample is better than that of high-temperature ceramics such as HEB, especially at temperatures approaching 1300 °C.

Vickers hardness measurements were performed using a Phase II+ 900-390A Micro Vickers Hardness tester (TEquipment, Long Branch, NJ, USA). The measured hardness data for the B_4,55_C sample at various loads with a dwell time of 15 s is shown in Table 1. Our measured value of hardness of 33.8 GPa at a maximum load of 200 gf is slightly higher than the 28–30 GPa values reported for various boron-rich boron–carbide materials [14] and could be related to differences in the microstructure of synthesized materials.

## 4. Discussion

Our direct synthesis of boron-rich boron–carbide material using spark plasma synthesis at high pressure and high temperatures indicates the stability of a rhombohedral phase with a composition of B_4.55_C (18 atomic percent carbon). These results are consistent with the fact that a single rhombohedral phase is stable between the range of B_10.4_C (8.8 atomic percent carbon) and B_4_C (20 atomic percent carbon). The X-ray photoelectron spectroscopy measurements reveal the characteristic binding energy of 188.5 eV for a boron–carbon bond established between the carbon and boron precursors during the high-pressure, high-temperature sintering process. The high hardness value of 33.8 GPa for B_4.55_C material is consistent with it being the third hardest material behind cubic boron nitride and diamond. Raman spectroscopy of B_4.55_C material documented the presence of an additional vibrational mode at 386 cm^−1^ that is attributed to excess boron that replaces carbon in the linear chain C-B-C to C-B-B. In addition, excess boron results in the distortion of the breathing mode of the boron icosahedron and results in a new Raman mode at 1161 cm^−1^. The thermal oxidation studies in compressed dry air on B_4.55_C material show less mass gain per unit area at 1300 °C as compared to the high-entropy transition metal boride, establishing it as a prime candidate for applicability in extremes of pressure, temperatures, and oxidizing environments. Future studies should aim at extending the thermal oxidation resistance of boron-rich boron–carbide materials to higher temperatures of around 2000 °C.

## 5. Conclusions

We report on the direct synthesis of boron-rich boron–carbide B_4+δ_C material using a ball-milled mixture of graphite and boron powders by spark plasma sintering at a pressure of 7 MPa and at a temperature of 1930 °C. The synthesized material is a rhombohedral phase with measured lattice parameters in hexagonal representation of *a* = 5.609 ± 0.007 Å and *c* = 12.082 ± 0.02 Å, and comparison with published trends shows the composition to be B_4.55_C. X-ray photoelectron spectroscopy reveals a B-C bond with a binding energy of 188.5 eV, and Vickers hardness yields a high hardness value of 33.8 GPa. Raman spectroscopy has identified a new vibrational mode at 386 cm^−1^ representative of C-B-B linear chain formation due to excess boron in the lattice. There is an additional vibrational mode at 1161 cm^−1^ that can be attributed to the distortion of the breathing mode associated with the boron icosahedron. The quantitative thermal oxidation in compressed dry air indicates that B_4.55_C material has a better oxidation resistance behavior at 1300 °C compared to high-temperature ceramics such as the high-entropy transition metal borides. The high neutron absorption cross section, high melting point, high mechanical strength, and thermal oxidation resistance make the boron-rich boron–carbide material ideally suited for applications in extreme environments.

## Figures and Tables

**Table 1:** Measured Vickers hardness for the B_4,55_C sample at various applied loads with dwell times of 15 s each.

Applied Load in g	Vickers Hardness (GPa)
50 gf	39.2 ± 5.1 GPa
100 gf	37.0 ± 0.8 GPa
200 gf	33.8 ± 2.3 GPa

## Data Availability

All data generated or analyzed, and materials synthesized during this study are included in this published article. Additional raw data used and/or analyzed during the current study are available from the corresponding author on reasonable request.
